# Hospital-level associations with 30-day patient mortality after cardiac surgery: a tutorial on the application and interpretation of marginal and multilevel logistic regression

**DOI:** 10.1186/1471-2288-12-28

**Published:** 2012-03-12

**Authors:** Masoumeh Sanagou, Rory Wolfe, Andrew Forbes, Christopher Michael Reid

**Affiliations:** 1 Department of Epidemiology and Preventive Medicine, Faculty of Medicine, Nursing and Health Sciences, Monash University, Melbourne, Australia

## Abstract

**Background:**

Marginal and multilevel logistic regression methods can estimate associations between hospital-level factors and patient-level 30-day mortality outcomes after cardiac surgery. However, it is not widely understood how the interpretation of hospital-level effects differs between these methods.

**Methods:**

The Australasian Society of Cardiac and Thoracic Surgeons (ASCTS) registry provided data on 32,354 patients undergoing cardiac surgery in 18 hospitals from 2001 to 2009. The logistic regression methods related 30-day mortality after surgery to hospital characteristics with concurrent adjustment for patient characteristics.

**Results:**

Hospital-level mortality rates varied from 1.0% to 4.1% of patients. Ordinary, marginal and multilevel regression methods differed with regard to point estimates and conclusions on statistical significance for hospital-level risk factors; ordinary logistic regression giving inappropriately narrow confidence intervals. The median odds ratio, MOR, from the multilevel model was 1.2 whereas ORs for most patient-level characteristics were of greater magnitude suggesting that unexplained between-hospital variation was not as relevant as patient-level characteristics for understanding mortality rates. For hospital-level characteristics in the multilevel model, 80% interval ORs, IOR-80%, supplemented the usual ORs from the logistic regression. The IOR-80% was (0.8 to 1.8) for academic affiliation and (0.6 to 1.3) for the median annual number of cardiac surgery procedures. The width of these intervals reflected the unexplained variation between hospitals in mortality rates; the inclusion of one in each interval suggested an inability to add meaningfully to explaining variation in mortality rates.

**Conclusions:**

Marginal and multilevel models take different approaches to account for correlation between patients within hospitals and they lead to different interpretations for hospital-level odds ratios.

## Background

Over the past two decades there has been a dramatic growth in the publication of cardiac surgery outcomes research. Many recent studies have examined the impact of hospital, physician, and process-related characteristics on outcomes for hospitalized patients who have undergone cardiac surgery [1-8]. By virtue of their observational design, these studies rely heavily on the use of regression modelling to remove the effects of confounding variables [9-11]. Data from these studies usually have a two-level structure of patients within hospitals, a familiar structure in epidemiological studies [12,13].

It is generally recognised in many areas of the social, medical and other sciences that data arise in complex multilevel structures, for example responses from individuals who are grouped together in communities or institutions. An understanding of appropriate analytical methods is vital for researchers in fields such as education, epidemiology, geography, child growth and social surveys, among others. There is a rich literature on analytical methods for two-level data structures with particular emphasis on multilevel [14-17] and marginal [14,17] models. These methods have subtle differences in interpretation when applied in different multilevel contexts, for example to longitudinal studies [18,19], to cluster randomised trials [20], or, as focussed on here, to observational studies in which individual responses are correlated due to a shared environment or process the nature of which may only in part be measurable.

Ordinary (single level) logistic regression is usually inappropriate for patient-within-hospital outcomes because it assumes all outcomes are independent [21,22]. Patients within a given hospital typically tend to be more alike than patients across different hospitals in measured and unmeasured characteristics predictive of outcome, for example socio-economic status. Further, at the hospital-level, the implementation of specific quality assurance programs (such as treatment protocols and critical care maps) may result in less heterogeneity in the use of evidence-based therapies for patients admitted to a particular hospital [23]. Ignoring the clustering present in multilevel data, as occurs in ordinary logistic regression, results in an artificially inflated number of independent observations at the hospital level of the hierarchy, and hence is likely to underestimate the magnitude of the standard error for the effect of hospital-level characteristics. Marginal and multilevel statistical techniques have been developed to deal with data arranged in a natural hierarchy [15,16,24]. Ordinary and multilevel models have been used to investigate whether the choice of statistical methods affects which hospitals are classified as high- and low-performance outliers in the publicly available data at the New York State, Department of Health, Coronary Artery Bypass Graft (CABG) Surgery Report Card [25]. In the context of patients-in-hospitals two-level data, marginal [26] and multilevel logistic regression [27,28] have been used to examine the impact of risk factors on outcomes when adjusting for differences in patient and hospital characteristics. The interpretation of effect estimates differs for marginal and multilevel models for binary outcomes and this is understood when interpreting patient-level risk factors [29,30]. Not so widely appreciated is how the interpretation of hospital-level effects also differs between these models. Further, multilevel logistic regression offers additional parameters, the median odds ratio (MOR) [31,32], and 80% interval odds ratios (IOR-80%) [31,32], that can help to shed light on hospital-to-hospital variability in outcome and the impact of hospital-level risk factors, respectively.

The aim of this study was to apply ordinary, marginal and multilevel logistic regression models to 30-day mortality outcomes of 32,354 patients in the Australasian Society of Cardiac and Thoracic Surgeons (ASCTS) registry in order to compare the three methods with the focus on the interpretation of hospital-level risk factors.

## Methods

### Patient population and data

The Australasian Society of Cardiac and Thoracic Surgeons (ASCTS) is responsible for a registry of cardiac and thoracic surgery in Australia covering nearly half of the country's private and public cardiothoracic surgical units.

Data was collected on all patients undergoing cardiac surgery between July 2001 and June 2009 in 18 hospitals in Australia. In each hospital, a data manager was responsible for the completeness of the data collection. All data was verified on receipt by the co-ordinating centre which followed up on queries about missing data, outliers or inconsistent reports. The data was validated locally and also by an external data quality audit program. This program was performed on-site to evaluate the completeness (2.4% missing value) and accuracy of the data collected within the combined database [33].

The ASCTS registry collected information on patient preoperative risk factors (including preoperative cardiac status and previous interventions), intra-operative details (including the procedure performed, myocardial protection and procedural duration), complications and post operative outcomes. In this study, the outcome variable was mortality within 30-days of cardiac surgery. This information was collected by the data managers by contacting medical practitioners, patients or family members by telephone as part of clinical care.

This research project was undertaken following approval from the ASCTS Research Committee which governs access to data from the registry. Ethical approval for the use of de-identified registry data for secondary research purposes such as this project had previously been provided by each participating institution's ethics review committee.

### Patient and hospital level characteristics

Patient-level characteristics in the registry included: age, gender, the New York heart association (NYHA) class, urgency of procedure, ejection fraction estimate, lipid-lowering treatment (hypercholesterolaemia), preoperative dialysis, previous cardiac surgery, procedure type, inotropic medication (inotropes), peripheral vascular disease, and body mass index (BMI) [34]. Two hospital-level characteristics were assessed, namely academic affiliation (teaching or non-teaching status) and the median across 2001-2009 of the annual number of cardiac surgeries. Academic affiliation of the 15 teaching hospitals and 3 non-teaching hospitals did not change throughout the study period. All patient and hospital characteristics were included in analysis as categorical risk factors except the median annual number of cardiac surgeries which was continuous.

### Statistical techniques

#### Ordinary, marginal and multilevel logistic regression

Logistic regression [35] was used to assess the influence of risk factors on 30-day mortality. Let p_ij _be the probability and $\frac{p_{ij}}{1-p_{ij}}$ the odds of death for patient j in hospital i. The equation of the ordinary logistic model was

(1) $$log\left(\frac{p_{ij}}{1-p_{ij}}\right)=\beta_{0}+\overset{R}{\underset{k=1}{\Sigma }}\;\beta_{k}X_{ijk}$$

where the X_ijk_'s represent a patient's values of R risk factors, and β_1_...β_R _are regression coefficients corresponding to each risk factor. For a given risk factor, its coefficient β_k _is the log odds ratio corresponding to a 1-unit difference in continuous X_k _or, if a risk factor is an indicator, for example of peripheral vascular disease (1 if yes, 0 if no), then β_k _is the log odds ratio comparing the effect on mortality of the risk factor's presence with its absence. Exponentiating β_k _($e^{\beta_{k}}$) gives the corresponding odds ratio, OR. In ordinary logistic regression hospital characteristics are treated the same as patient-level risk factors, and patient outcomes Y_ij _are assumed to be independent binomial variables with mortality probability p_ij._

A model based on generalized estimating equations, GEE [36], may also be used for analysis of patient mortality outcomes. For this, equation (1) is combined with the following assumptions: firstly as before the probability of Y_ij _= 1, a death, is p_ij _and Y_ij _has binomial variance p_ij_(1-p_ij_). Secondly, it is assumed that patients within a hospital have correlated outcomes but patient outcomes in different hospitals are independent, i.e. have zero correlation. An exchangeable working correlation structure in the GEE estimation process assumes that pair-wise correlations between patient outcomes within the same hospital are equal and can be represented by the parameter ρ.

The GEE method includes the calculation of robust estimates for the standard errors of the regression coefficients that ensure consistent inference even if the chosen working correlation structure is incorrect or if the strength of the correlation between patient outcomes within the same hospital varies somewhat from patient to patient.

Multilevel logistic regression [15] assumes that each hospital has its own underlying mortality probability and this varies from hospital to hospital. Specifically, a logistic regression for patients includes an additional term u_i_, a hospital-level random effect, as a predictor variable:

(2) $$log\left(\frac{p_{ij}^{*}}{1-p_{ij}^{*}}\right)=u_{i}+\beta_{0}+\sum_{k=1}^{R}\;\beta_{k}X_{ijk}$$

Note that $p_{ij}^{*}$ in this model is the conditional probability that patient j in hospital i died, and here the probability depends on the value of the random effect, u_i_, for that hospital. u_i _is the totality of measured and unmeasured hospital-level variables that predict mortality and are uncorrelated with the individual and hospital-level predictor variables in the model. In other words u_i _represents the combination of omitted hospital-level variables.

Variation in the mortality propensity between hospitals is accommodated by assuming a normal distribution for u_i _with mean zero and variance τ^2^. A hospital with u_i _= 0 can be thought of as having "average" (compared to other hospitals in the population) mortality probability (on the log odds scale). Higher values of τ^2 ^indicate greater heterogeneity in mortality among hospitals. By including u_i _in the model as a random effect, the interdependencies among patients within hospitals are explicitly taken into account.

#### Odds ratio interpretation in ordinary, marginal and multilevel logistic regression

The interpretation of the odds ratio for patient-level risk factors (e^β^) in ordinary logistic and marginal models is the same, but differs from the interpretation in the multilevel logistic model [29]. The marginal models estimate population-averaged (or population-marginalized) parameters [30]. In a marginal model, odds ratios characterize the effect of predictors on the population as a whole, averaged over u_i_, rather than on a typical hospital [30]. Odds ratios in a marginal model represent, across all hospitals, differences in mortality between all patients with one value of a risk factor to all patients with the other value.

In multilevel models, for patient-level variables, the usual odds ratio interpretations apply for comparisons of patients within the same hospital; for example, a body mass index (BMI) effect may be interpreted as an odds ratio between a patient with BMI < 25 and a patient with BMI > 25 belonging to the same hospital and with the same covariates, except for BMI.

Odds ratios for hospital risk factors in marginal models are interpreted as the odds of mortality for hospitals with one value of the factor compared to hospitals with another value of the factor. For example, the odds of 30-day mortality for non-teaching hospitals compared to teaching hospitals.

However, the odds ratio for a hospital-level risk factor in multilevel logistic regression has a different interpretation, namely the odds of mortality for hospitals with one value of the factor compared to hospitals with another value of the factor but with the same value of random effect. For example, the odds of 30-day mortality for a patient treated at a non-teaching hospital compared to a patient treated at a teaching hospital with the same value of u_i_. Because of this difficult interpretation an additional parameter, the 80% interval odds ratio (IOR-80%), has been developed and is described later in this paper.

The OR for a risk factor in a marginal model is adjusted for the other risk factors included in X_ijk_. In a multilevel model the ORs are additionally adjusted for unobserved hospital-level characteristics via the random effect. Due to a mathematical property called non-collapsibility of the odds ratio, the odds ratio for a risk factor from a multilevel model is likely to be further from the null value of one than the odds ratio for that risk factor from a marginal model [37].

#### Intra-class correlation coefficient (ICC)

The fundamental reason for applying special statistical techniques in multilevel analysis is the likely existence of intra-class (intra-hospital) correlation arising from similarity of mortality risk of patients of the same hospital compared to those of different hospitals. Patients operated on at the same hospital may be more similar to each other than patients operated on in other hospitals, as they share a number of economic, social, and other characteristics that may condition similar health status beyond what can be adjusted for by patient-level covariates.

The total variance in the outcome variable is the sum of patient-level and hospital-level variances. In multilevel logistic regression however, the patient-level variance is on the probability scale whereas the hospital-level variance is on the logistic scale. To solve this technical difficulty the linear threshold approximation has been proposed [16] and its solution is to convert the patient-level variance from the probability scale to the logistic scale. The method assumes that the propensity to die is a continuous latent variable and only those patients whose propensity crosses a certain threshold will die as defined by the binary outcome. The unobserved patient variable follows a logistic distribution with patient-level variance equal to 3.29. On this basis, the ICC is calculated as:

(3) $$ICC=\frac{\tau^{2}}{\tau^{2}+3.29}$$

where τ^2 ^is the estimated variance of the random effect of hospital.

In the marginal model, the working correlation structure is the mechanism that accounts for ICC, and the parameter ρ can be interpreted as a measure of intra-hospital correlation in mortality outcomes. There is a subtle distinction between ρ and the ICC defined in (3) for the conceptual propensity-to-die variable. It has been shown that ρ in a marginal model is smaller than ICC in the corresponding multilevel model when there are a small number of clusters [38].

For a binary outcome like mortality, the term ICC can be difficult. Conceptual problems with the ICC (it is a concept from linear regression that has no exact equivalent for logistic regression), interpretational issues as outlined above, and generalisability problems (ICC depends on outcome prevalence) are some of its limitations. Similarly, τ^2^, the inter-hospital variation in mortality, is difficult to interpret because it is on a log-odds scale [39,40].

#### The median odds ratio (MOR)

The MOR is potentially easier to interpret than the ICC because it expresses inter-hospital variance on the OR scale, on which the effects of risk factors are also interpreted.

MOR is defined for a multilevel model as the median of the set of odds ratios that could be obtained by comparing two patients with identical patient-level characteristics from two randomly chosen, different hospitals, i.e. different in hospital random effect value [31,32]. The MOR is the median odds ratio between the patient in the hospital with higher mortality propensity and the patient in the hospital with lower mortality propensity.

The MOR is a measure of variation between the mortality rates of different hospitals that is not explained by the modelled risk factors. The MOR can be shown [41] to be simply related to τ^2 ^as

(4) $$MOR=exp\left[\sqrt{2\times \tau^{2}\times 0.6745}\right]\approx exp\left(0.95\tau \right)$$

where τ^2 ^is the hospital-level variance. If the MOR is 1, there is no variation between hospitals. If there is considerable between-hospital variation, the MOR will be large.

Because the two measures of inter-hospital variation, τ^2 ^and ICC, are difficult to interpret [31] the MOR is considered as an alternative measure. While ICC and MOR have a direct relationship due to their shared basis on τ^2^, the different functions of that inter-hospital variation give rise to usefully distinct interpretations.

#### The 80% interval odds ratio (IOR-80%)

The interpretation of hospital-level effects in multilevel model has been highlighted as problematic. In multilevel models, contrary to patient-level risk factors, hospital-level risk factors only take one value in each hospital and, consequently, it is necessary to compare patients from different hospitals to quantify hospital-level associations with outcome [41,42]. A multilevel model odds ratio for a hospital-level risk factor needs to be interpreted as the effect of the risk factor given a comparison between two hospitals of identical random effect value whose mortality probabilities differ only with regard to the risk factor under consideration.

To interpret effects of hospital-level risk factors more generally, the unexplained between-hospital variability needs to be taken into account. The IOR-80% achieves this by incorporating both the fixed hospital-level risk factor effect and the unexplained between-hospital heterogeneity in an interval. IOR-80% shows the impact of hospital-level variables on mortality when comparing hospitals with different u_i _values.

To understand the calculation of the IOR-80%, consider all possible pairs of patients with identical patient-level risk factors from different hospitals but who differ by one unit in a hospital-level risk factor (e.g. one patient in a teaching hospital, the other in a non-teaching hospital). For each pair, the OR between the two patients is calculated, thereby obtaining a distribution of ORs. The IOR-80% is defined as the interval around the median of the distribution that comprises 80% of the OR values. In practice, the lower and upper bounds of the IOR-80% can be computed using the approximation

(5) $$IOR-80{\%}_{\frac{lower}{upper}}=exp\left[\beta \pm 1.2816\sqrt{2\times \left(\tau^{2}\right)}\right]\approx exp\left(\beta \pm 1.81\tau \right)$$

where β is the regression coefficient for the hospital-level variable, τ^2 ^is the hospital-level variance, and the values -1.2816 and +1.2816 are respectively the 10th and 90th centiles of the standard normal distribution.

From equation (5), a small amount of between hospital variation, τ^2^, will lead to a narrow IOR, whereas large τ^2 ^leads to wider intervals. The combination of τ^2 ^with the effect of the hospital-level risk factors in (5), indicates that the IOR-80% will contain 1 if the value of τ^2 ^is large compared to the effect of the hospital-level risk factors.

#### Model estimation

To fit the ordinary logistic regression model, maximum-likelihood was used with the Stata [43] (version 11) logistic command. The marginal logistic regression model was fitted with Stata's xtgee command and multilevel models were fitted using adaptive quadrature with 12 integration points to evaluate and maximize the marginal log likelihood by Stata's xtlogit command.

## Results

**Figure 1:**
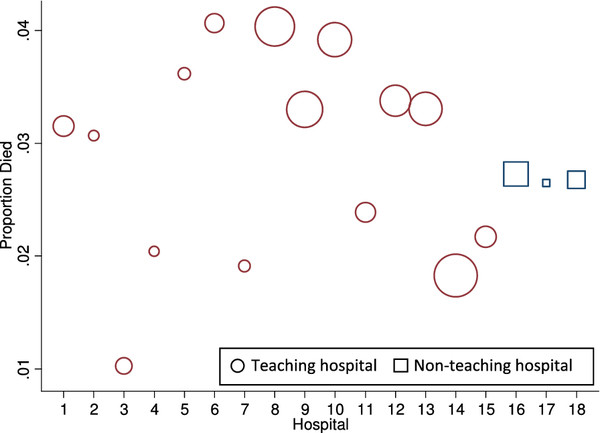
**Mortality within 30-days of cardiac surgery in teaching and non-teaching hospitals**. The area of their circle is proportional to the number of surgeries in each of the 18 hospitals.

The cohort consisted of 32,354 patients on the ASCTS registry admitted to 18 hospitals from 2001-2009. Patient mean age was 65.5 years (SD 12.5); 27.7% were female. Table 1 includes further patient characteristics. The number of cardiac surgeries ranged from 151 to 5314 across hospitals (Table 2). Figure 1 shows that variation in mortality rates across hospitals was considerable with rates in the range 1.0 to 4.1%.

**Table 1:** Patient characteristics from a cardiac surgery registry 2001-2009

Patient characteristics	Frequency	Percent
**Age (years)**		
< 60	8954	27.6
60 to 70	9434	29.2
70 to 80	10695	33.1
80+	3271	10.1
**Gender**		
Male	23385	72.3
Female	8969	27.7
**The New York heart association class**		
I&II	22470	69.4
III	7245	22.4
IV	2639	8.2
**Urgency of procedure**		
Elective	19869	61.4
Urgent	10665	33.0
Emergency/salvage	1807	5.6
**Ejection fraction estimate (%)**		
Mild (> 45)	21919	80.0
Moderate (30-45)	3828	14.0
Severe (< 30)	1656	6.0
**Hypercholesterolaemia**	22072	68.6
**Preoperative dialysis**	523	1.6
**Previous cardiac surgery**	2776	8.6
**Procedure type**		
CABG	19907	61.6
Valve(s)	4571	14.1
Valve(s)+CABG	3367	10.4
Other	4472	13.8
**Inotropic medication**	888	2.8
**Peripheral vascular disease**	3583	11.1
**Body mass index (kg/m^2^)**		
< 25	9077	28.1
25+	23277	71.9

**Table 2:** Total number of cardiac surgeries, median of the annual number of cardiac surgeries, number of deaths within 30-days of surgery, and mortality rates in 18 hospitals 2001-2009 by academic affiliation status

Hospital	The median annual number of cardiac surgeries	No. of surgeries*	No. of deaths	Mortality rate (%)
**Teaching**				
1	310	1174	37	3.15
2	163	326	10	3.07
3	195	780	8	1.03
4	147	294	6	2.04
5	147	470	17	3.62
6	293	1083	44	4.06
7	209	418	8	1.91
8	512	4361	176	4.04
9	437	3729	123	3.30
10	418	3291	129	3.92
11	284	1131	27	2.39
12	332	2753	93	3.38
13	402	3206	106	3.31
14	649	5314	97	1.83
15	324	1291	28	2.17
**Non-teaching**				
16	475	1723	47	2.73
17	75	151	4	2.65
18	172	859	23	2.68

* Not all hospitals reported for all 9 years in the period 2001-2009

**Figure 2:**
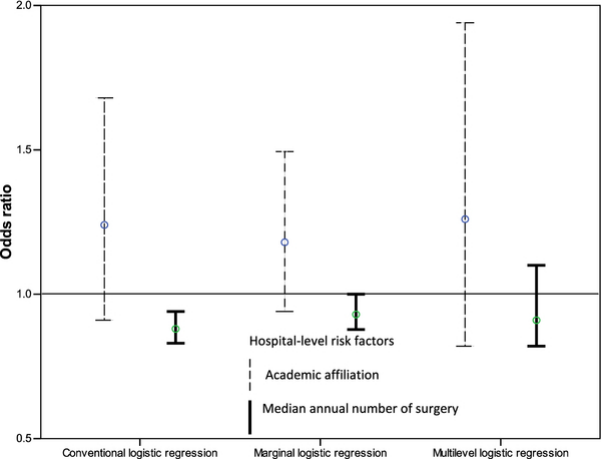
**Odds ratio estimates and 95% CIs derived from conventional, marginal and multilevel logistic regression for 30-day mortality for academic affiliation and median annual number of surgery**.

Table 3 contains the odds ratios and 95% CIs for the effects of patient and hospital characteristics from the ordinary, marginal and multilevel logistic regression models. Overall the 95% CIs for hospital-level variables in the marginal and multilevel model were wider than in the ordinary logistic regression, reflecting the between-hospital heterogeneity that is erroneously not accounted for in the latter model (Figure 2). In particular the effect of median annual number of cardiac surgeries was statistically significant in ordinary logistic regression, but not in the other models.

**Table 3:** Ordinary, marginal and multilevel logistic regression results: Odds ratios, OR, describing associations with 30-day mortality for patient-level and hospital-level characteristics

Risk factor	Ordinary		Marginal		Multilevel*	
	
	OR	95% CI	OR	95% CI	OR	95% CI
**Patient-level**						
**Age group (< 60 y, reference group):**						
60-70 y	1.5	(1.2,2.0)	1.5	(1.2-1.8)	1.5	(1.2,2.0)
70-80 y	2.7	(2.1,3.4)	2.4	(2.0-3.0)	2.7	(2.1,3.3)
80+y	3.6	(2.8,4.8)	3.3	(2.7-4.5)	3.7	(2.8,4.9)
**Gender (female vs. male)**	1.3	(1.1,1.6)	1.3	(1.1-1.5)	1.3	(1.1,1.6)
**NYHA Class (I&II, reference group):**						
III	1.3	(1.1,1.6)	1.3	(1.1-1.6)	1.4	(1.1,1.6)
IV	1.7	(1.4,2.1)	1.6	(1.3-2.0)	1.7	(1.4,2.2)
**Urgency of procedure (elective, reference group):**						
Urgent	2.0	(1.6,2.4)	1.8	(1.5-2.2)	2.0	(1.6,2.4)
Emergency/salvage	5.4	(4.3,7.0)	4.9	(4.0-6.0)	5.5	(4.3,7.0)
**Ejection fraction estimate (Mild, > 45%, reference group):**						
Moderate (30-45%)	1.4	(1.2,1.8)	1.3	(3.0-1.6)	1.4	(1.2,1.8)
Severe (< 30%)	2.3	(1.8,2.9)	2.2	(1.8-2.7)	2.3	(1.8,2.9)
**Hypercholesterolaemia**	1.0	(0.8, 1.1)	1.0	(0.8-1.1)	1.0	(0.8,1.2)
**Preoperative dialysis**	2.2	(1.5,3.2)	2.2	(1.5-3.0)	2.2	(1.5,3.2)
**Previous cardiac surgery**	1.6	(1.3,2.0)	1.6	(1.3-2.0)	1.7	(1.3,2.0)
**Procedure type (CABG, reference group):**						
Valve(s)	1.7	(1.3, 2.2)	1.6	(1.2-2.0)	1.7	(1.3, 2.2)
Valve(s)+CABG	2.2	(1.8, 2.8)	2.0	(1.6-2.6)	2.2	(1.8, 2.8)
Other	3.4	(2.7,4.1)	3.0	(2.4-3.7)	3.4	(2.8,4.2)
**Inotropic medication**	3.1	(2.4,3.9)	3.0	(2.4-3.7)	3.2	(2.5,4.1)
**Peripheral vascular disease**	1.7	(1.4,2.1)	1.6	(1.3-2.0)	1.7	(1.4,2.0)
**Body mass index (25+ vs. < 25 kg/m^2^)**	0.8	(0.7,1.0)	0.9	(0.7-1.0)	0.8	(0.7,1.0)
**Hospital-level**						
**Academic affiliation (teaching vs. non-teaching)**	1.2	(0.9,1.7)	1.2	(0.9-1.5)	1.3	(0.8,1.9)
**Median of the annual number of cardiac surgeries 2001-2009, per 100 surgeries**	0.88	(0.83,0.94)	0.93	(0.9-1.0)	0.91	(0.82,1.1)

*The estimated hospital-level variance, τ^2^, was 0.04.

*The estimated hospital-level variance, τ^2^, was 0.04

In the marginal model the correlation between mortality outcomes for any two patients from the same hospital was ρ = 0.002 suggesting a weak positive association. The multilevel model estimated that the proportion of the variance in 30-day mortality between hospitals was 1% (ICC = 0.01). From the multilevel model it was estimated that if a patient moved to another hospital with a higher probability of mortality, the median increase in their odds of mortality would be 1.2-fold (MOR = 1.2), a modest effect compared to patient-level risk factor effects in Table 2 but comparable to the hospital-level fixed effects in Figure 2.

Interpretation of the hospital-level effects estimated from marginal logistic regression (OR = 1.2, 95%CI, (0.9-1.5))was that, on average, the odds of mortality for patients in teaching hospitals increased by 20% compared to that of patients in non-teaching hospitals. In comparison, the multilevel logistic regression odds ratio of 1.3, 95%CI, (0.8-1.9) for the same parameter says that if comparing two patients with identical risk factors, one treated in a teaching hospital and one treated in a non-teaching hospital, and with those hospitals otherwise identical with regard to mortality risk, then the odds of mortality was increased 1.3-fold for the patient in the teaching hospital. The magnitude of these effects may be of high importance clinically but the difficulty in interpretation, particularly with regard to the existence of hospitals with identical underlying mortality risk, may limit their usefulness.

The IOR-80% for academic affiliation was 0.8 to 1.8 which provides the further insight that, when comparing two randomly chosen patients with identical risk factors, one from a teaching hospital, the other from a non-teaching hospital, and those hospitals possibly differing in other ways regarding mortality risk, the odds ratio for the comparison will, with 80% probability, lie between 0.8 and 1.8. In other words, even disregarding the uncertainty inherent in sampling that can be incorporated in confidence intervals, the wide IOR-80% reflects considerable uncertainty in the impact of hospital academic affiliation on patient-level mortality risk due to substantial residual variation in mortality between hospitals that was not accounted for by either academic affiliation or median annual number of cardiac surgeries or patient-level characteristics included in the regression model.

The IOR-80% for the median of the annual number of cardiac surgeries was 0.6 to 1.3. Hence when comparing two randomly chosen patients with identical risk factors except for treatment at respective hospitals which differed by 100 in their median annual number of cardiac surgeries, and possibly differing in u_i _values, the odds ratio for the comparison will, with 80% probability, lie between 0.6 and 1.3. As for academic affiliation, this is a wide IOR-80%.

## Discussion

This paper examined the application and interpretation of ordinary, marginal and multilevel logistic regression for explaining between-hospital heterogeneity in 30-day mortality outcomes following cardiac surgery in Australian hospitals.

While this paper focused on the three measures, ICC, MOR and IOR_80%, there are other measures of variance and clustering. Alternating logistic regression [44], ALR, is a method for a statistical index of patient clustering in the form of pair-wise odds ratio. The proportional change in variance [45] is another measure that could be used for explaining variance across hospitals by patient characteristics. While we interpret ρ as an estimate of ICC, this correlation between pairs of binary outcomes can be of particular interest in twin or longitudinal data [46]. The relative strengths of ALR, multilevel and marginal logistic regression have been examined by others [47].

Appropriately reflecting the between-hospital heterogeneity, the 95% confidence intervals for hospital-level variables in the marginal and multilevel model were wider than in the ordinary logistic regression. The MOR translated the impact of the between-hospital residual variability to an effect describing relative mortality risk of patients form different hospitals. The IOR-80% for both academic affiliation and median annual of cardiac surgeries were relatively wide, reflecting the large unexplained variation between hospitals in mortality. The inclusion of OR = 1 in both intervals suggested an inability of hospital-level risk factors to add meaningfully to explaining variation in mortality rates.

Marginal models do not require any distributional assumptions beyond correctly modelling the mean (average) outcome, yet when combined with robust standard errors they can provide appropriate inferences. They estimate within-hospital dependency, but do not directly estimate variance components. A methodological limitation of relevance here is that the robust standard errors are underestimated by marginal models for studies with a small number of hospitals, especially if the numbers of patients per hospital are severely unbalanced. Using bootstrap methods for estimating standard errors is an alternative approach [41,48-50].

In contrast, the most straightforward interpretation of hospital to hospital variability in mortality came from the MOR in the multilevel model. Being on the odds ratio scale, the MOR allows unexplained hospital to hospital variability to be directly compared with patient-level and hospital-level risk factor effects. Using IOR-80% for measuring the association between the hospital-level risk factors and mortality, the between hospital heterogeneity becomes relevant for understanding the real impact of hospital-level risk factors.

In previous studies, for assessing hospital-level characteristics on mortality, ordinary and multilevel logistic regression were compared but ICC, MOR and IOR-80% were not reported,[51,52] or only ICC provided [23].

Multilevel and marginal methods address different questions and the choice of method needs to be made according to the research objective. Multilevel models are best equipped to address questions relating to modification of a particular hospital [29]. A marginal approach does not make specific use of within-hospital information for hospital-level covariates [29]. For example, in a study assessing hospital-level characteristics on mortality after acute myocardial infarction the OR point estimates were of interest more than their statistical significance, so an ordinary logistic regression model was applied for its greater familiarity and hence comprehensibility [53]. The marginal model is recommended if the aim is estimation of the effects of patient-level risk factors while adjusting for between hospital heterogeneity. Marginal logistic regression with the exchangeable working correlation matrix has been used for patients within surgical centres [26] where the study goal was to determine the relationship between in-hospital mortality after coronary artery bypass graft surgery.

For specific hospital interpretation or hospital ranking the multilevel model is recommended [54,55]. In a study for comparing neonatal mortality in low- and high-risk deliveries in different hospitals MOR and IOR-80% were reported [56] however use of these two statistics has not extended to cardiac surgery outcome analysis.

## Conclusion

This paper outlines the application and interpretation of marginal and multilevel modelling to patient-level cardiac surgery outcomes observed across multiple hospitals. The interpretation of hospital-level risk factors differs between methods and the subtleties of the interpretations have been clarified.

Choosing between a marginal or multilevel model depends on the goals of the analyses. Knowing the assumptions of each method and how these assumptions affect the inferences from the analysis will enable researchers to determine the best approach to analysing their data.

## Competing interests

The authors declare that they have no competing interests.

## Authors' contributions

MS designed and conducted the study, performed the statistical analysis, wrote the draft of the manuscript to which all authors subsequently contributed. RW and AF contributed to the design and conduct of the study CMR was responsible for the data collection process and issues related to data quality. All authors read and revised the manuscript for important intellectual content and approved the final manuscript.

## Pre-publication history

The pre-publication history for this paper can be accessed here:

http://www.biomedcentral.com/1471-2288/12/28/prepub
